# Attachment Styles in Children Living in Alternative Care: A Systematic Review of the Literature

**DOI:** 10.1007/s10566-015-9342-x

**Published:** 2015-12-17

**Authors:** Manuela Garcia Quiroga, Catherine Hamilton-Giachritsis

**Affiliations:** School of Psychology, University of Birmingham, Edgbaston, Birmingham, B15 2TT UK; Department of Psychology, University of Bath, Claverton Down, Bath, BA2 7AY UK

**Keywords:** Attachment, Alternative Care, Institution, Foster care, Children’s Homes, Caregivers

## Abstract

**Background:**

A large number of children are currently living in Alternative Care. The relationship they establish with their temporary caregivers can play a significant role in their development. However, little has been published regarding attachment with *temporary Caregivers*.

**Objective:**

The aim of this review is to analyse the existing published studies regarding attachment styles in children *living in alternative care* (Children’s Homes and Foster Care). The review analyses rates of attachment styles and associated factors (including characteristics of settings, children and caregivers) in both settings.

**Methods:**

A systematic literature review was conducted searching electronic databases for peer reviewed publications in different languages. Studies considering attachment in children living in Children’s Homes or Foster families at the time of the study were included.

**Results:**

Overall, 18 articles reporting 13 studies met the inclusion criteria. The results are presented in terms of characteristics of the studies, rates of attachment in different settings and possible mediating factors. Implications for practice and research are discussed.

**Conclusions:**

Attachment styles in children living in alternative care differ from those observed in children living with biological or adoptive families, however several factors can mediate this outcome (including characteristics of settings, children and caregivers). Most research has been conducted in Europe and USA. Therefore, further research is needed in less developed countries in order to guide local policies for better care.

## Introduction

The importance of Attachment in children’s development has been widely studied and there is strong evidence about the impact of the relationship a child establishes with his primary caregivers on different developmental areas (i.e., cognitive, physical, emotional and social; Main et al. 1985; Sroufe 2005). Whilst the study of attachment was initially centred on the mother–child bond (Bowlby 1979), it was later developed to include the concept of multiple attachments, such as with the father, kin and day carers (Rutter et al. 2007; Santelices and Pérez 2013). This is particularly important to consider for orphans, abandoned children and those who are removed from their families for protection or other reasons (such as poverty, gender, disability or age of mother in different countries) and are taken into some form of ‘Alternative Care’ (AC)—either in Children’s Homes or foster families. The relationship that these children establish with their temporary caregivers has the potential to perpetuate or change previous attachment patterns. Yet, despite the importance of these relationships, only more recently have studies in attachment considered samples of children living in Children’s Homes or foster families *when* the studies were conducted. Given the likely impact of these relationships with Caregivers, having a clear understanding of these attachments and the factors that might impact upon them seems to be very important.

### Alternative Care

As well as those children without parents, an important number of children around the world have been removed from their families for several reasons, often for protection but also sometimes due to social or economic factors (E. C. Daphne Programme 2005). These children may be placed in Children’s Homes or foster families for different lengths of time before being adopted, returned to their biological families or even staying in Alternative Care until they reach adulthood.

The negative impact of institutional care on future development has been widely studied, with this impact shown to be stronger in the first 3 years of life (see Hamilton-Giachritsis and Garcia Quiroga 2014, for an overview of Institutional care). International recommendations on AC (United Nations, Guidelines for the Alternative Care of Children 2009) highlight the need to close institutions and develop foster care programs. However, whilst this process has begun in many countries, the implementation has been complex and several studies have revealed important difficulties with the placement of children in foster care, such as lack of motivation to foster due to cultural reasons, difficulties in supervision and support for foster parents leading to breakdowns and instability in placements and the overwhelmed foster care systems (Maluccio et al. 2006; Mapp 2011; UNICEF 2010).

Whilst in an ideal world institutional care would be phased out entirely, worldwide rates of child family maltreatment, street children and those being exploited, combined with children orphaned due to wars, natural disasters and health epidemics makes it difficult to find good quality family care for every child. Thus, the most probable scenario is that Children’s Homes will continue to exist in some form and it is very important that the environment to which children and youth are moved is significantly better that the environment from which they are removed. Although good quality and stable foster care would be preferred and should continue to be strived for, *in the absence of these*, protection needs to be effectively provided by good quality Children’s Homes, utilising research knowledge about how to make these environments as conducive to good child development as possible. For example, despite a lot of negative outcomes for children living in institutional care being identified in Europe (Johnson et al. 2006), in other parts of the world, children and young people have been shown to have good outcomes following institutional care. One study conducted in five less wealthy nations described no differences in health, emotional/cognitive functioning and physical growth outcomes for Orphans and Abandoned children living in institutional and community-based care (Whetten and the POFO Research Team 2009). Alongside other factors that might impact, it is useful to consider the role of attachment with alternative carers and the impact on likely prognosis and development.

### Attachment in Alternative Care

The relationship that children living in alternative care establish with their temporary caregivers has the potential to either perpetuate or change the previous patterns of attachment the child had built up with prior caregivers (biological parents or other previous placements). In alternative care, children also need to process their losses and previous traumatic experiences; thus, an adequate and sensible caregiver can become a secure base to the child in order to build up a relationship that can help in this process. Potentially, having the experience of a secure attachment can lead the way to future positive attachments with adoptive or biological parents. Yet attachment between the child and the caregiver is often discouraged as a way to “protect” the children from the pain of future separations, thereby limiting the possibility of change in the internal working models of these children.

In 1999, Smyke, Dumitrescu and Zeanah conducted a study in a Romanian institution with three groups: (a) a ‘typical’ unit; (b) a pilot unit with fewer adults caring for each child, giving greater stability in care; and (c) a control group of never institutionalised children. They found significantly higher rates of Reactive Attachment Disorder (RAD) in children in the typical unit than in the other two groups. Notably, children described as ‘their favourite’ by a caregiver had lower rates of attachment disorders (Smyke et al. 2002).

On a positive note, the St. Petersburg-USA Orphanage Intervention Study (2008) found that improvements in institutional care can have a significant impact on a wide range of areas of development, including child–caregiver relationship and attachment. An intervention based on structural changes (smaller groups and fewer changes of caregivers) and training (with a socio-emotional perspective) proved to have a wide impact on children’s’ development. Similarly, two intervention studies in Latin America found that staff training led to an improvement in caregiver–child interactions, with warmer and sensitive response impacting positively on children’s development (Lecannelier et al. 2014; McCall et al. 2010). Hence, the importance of child–caregiver interactions is clear.

An interesting review by Bakermans-Kranenburg et al. (2011) looked at attachment and emotional development in institutional care, and included studies both with children living in institutions and post adoption studies. The authors underlined the importance of considering some specifics when studying attachment in these contexts. In particular, they highlighted the need to take into account the possible lack of a specific attachment in some children reared in institutions due to limitations in developing a stable relationship with a specific Caregiver, where this lack of attachment formation can be misunderstood as disorganised attachment (e.g., with the Strange Situation Procedure. They propose the use of an attachment formation rating scale in these context. The review also discusses the concept of indiscriminate friendliness, and the nature of it in institutional settings, stating that it may respond to different factors than those observed in family contexts. The authors highlight the need for further study considering quality of care at the micro caring environment.

However, although the Bakermans-Kranenburg et al. (2011) review did include some important studies of children within institutions, its main focus was the analysis of methodological issues regarding the assessment of attachment disorders, indiscriminate friendliness and attachment formation in these settings, as well as the development of attachment following adoption. Thus, it did not analyse rates of attachment styles found in studies conducted while children were still living in residential settings, and it includes both studies of institutionalised and post adoption children but no study of foster care. Its main aim was to discuss emotional development in institutional care or post adoption.

In summary, little has been published regarding studies with a focus on rates of attachment styles (secure, avoidant, anxious and disorganised) in children *living* with their temporary caregivers at the time of the study. Temporary (paid) caregivers are likely to differ significantly to those who chose to adopt a child from an institution, but have a key role to play in enabling a child’s recovery. In summary, the fact that most studies and reviews include post-adoption samples as well as children living in institutions makes it difficult to describe the specific relationship children establish with their temporary caregivers, as opposed to adoptive parents.

### Objectives

Therefore, this review aims to describe and analyse the research that has been published regarding studies of attachment styles with children living in foster care or Children’s Homes. It is the first review with a focus on *attachment to temporary caregivers* exclusively considering studies of attachment styles with children living in alternative care at the time of the study. Specifically, a comparison between two different types of AC settings (Institutional and Foster Care) is made. This is considered an important point as many countries are moving from institutional care to foster care. The review includes rates of attachment and aims to provide an integrated analysis of different factors affecting the quality of attachment with caregivers in AC settings. It also provides a critical review of methodological issues and suggestions about future research on this topic. This review considers studies conducted from 1987 to 2013, in order to evaluate developments in the research. The specific hypotheses to be considered were:

1. There will be differences in the attachment styles of children living in biological families, institutional and foster care respectively.
2. Children living in foster care will have more positive attachment representations compared to children still living in institutional care.
3. In both institutional settings and foster homes, the quality of attachment (i.e., security) will be related to a number of mediating factors, including higher sensitivity of caregiver, higher quality of caregiving, younger age at placement and motivations of caregiver.
4. There will be differences between countries and between different types of institutions and foster care programs, regarding rates of attachment styles.
5. Methodological challenges in the study of attachment in alternative care contexts will also be reviewed.

## Method

### Design

A standard Systematic Literature Review methodology was employed. This included a search strategy based on inclusion and exclusion criteria according to population, exposure, comparator and outcomes (PECO), followed by Quality Assessment (QA) according to the type of study (case–control, cross sectional, randomised control trial or longitudinal). QA criteria looked for *selection bias*, *performance and assessment bias*, and *attribution bias* (coding strategy: yes = 2, partly = 1 and no = 0). When the item was coded as unsure, more information was searched for (i.e., additional information not reported in the articles but stated in other publications and contacts with the authors when possible), to gain the final QA score.

### Search Strategy

The search of published articles was conducted with different databases (PsycInfo 1987–2013, Medline 1996–2013, Web of Science, ASSIA, Scielo, ChildLink!, Embase 1996–2013). The following search terms (with appropriate Booleans and truncations, plus English and American spellings) were used: attachment, attachment behaviour, attachment theory, attachment disorders, attachment style, attachment representations, bonding, foster children, foster care, foster parents, alternative care, out of home care, residential care, institutional care, abandoned children, children’s homes, family-type home and orphanages.

Different languages were included in the search (English, French, Portuguese and Spanish articles were considered). Experts were contacted for suggestion on relevant articles in the topic. In addition, a search for grey literature on the web was conducted and the reference lists of relevant articles were hand checked. The inclusion criteria considered:

- Population: Children aged 0–17 years
- Exposure: Children living in alternative care (institutions and foster families) at the time of the study for a minimum of 2 months.
- Comparator: General population 0–17 or no comparison group.
- Outcome: Measures of attachment styles in children living in Alternative Care.

The exclusion criteria were: studies of adoption, studies of adulthood after AC, studies of specific psychopathologies (i.e., Autism, special needs, developmental problems, prenatal exposure to drugs), studies of children previously institutionalised or fostered but then with adoptive or birth families, studies measuring attachment only in carers and studies that evaluate the impact of specific interventions (other than when the intervention is placement in a Foster Care Program). This review focused on empirical papers, therefore well-known reviews were not included (e.g., van den Dries et al. 2009).

This search generated a total of 634 articles. Following the inclusion criteria and after removing duplicates, 147 articles remained based on the title. A further 112 were excluded based on the abstract, leaving 35 to be read in full, of which 17 were excluded. Thus, 18 articles were selected for the literature review, which reported on data from 13 studies.

### Quality Assessment and Inter-Rater Reliability

All the articles had a QA score of 50 % or more, with the majority of them having 70 % or more. A decision was made to include all of them in the review in order to better represent all the different studies in the topic and to be able to give a more culturally diverse view of existing research. For inter-rater reliability, 20 % of the articles were double coded (cronbach alpha = .967); differences between coders were discussed and a consensus reached.

### Ethics Statement

This study does not include primary data, thus, no ethics approval was applicable. There are no conflict of interest present in this review.

### Conflict of Interest

The authors have no conflict of interest.

### Access to Data

The first author takes responsibility for the integrity of the data and the accuracy of the data evaluation and analysis.

## Results

### Description of the Studies

The 18 articles reviewed were based on **13 studies**. Two studies (The Bucharest Early Intervention Project [BEIP] and Cole) were reported in several different articles considering different topics with the same sample, sub-samples or at follow-up (see Table 1). The **location** of the studies varies; five of the 13 studies were conducted in the USA, four in European countries (France,^1^ Greece, Romania and Ukraine), two in Asia (Japan and Israel), one in Canada and one in Africa (D. R. Congo). None of the data of children living in AC (institutions or foster families) was collected in Latin America. Regarding the **settings,** six studies were conducted with children living in institutions and six of them with children living in Foster Care. Only one study considered samples in both institutions and foster care (McLaughlin et al. 2012) and, in that case, the Foster Care program was especially designed for the study.

**Table 1:** Description of the key methodologies in the studies (N = 18)

Study	Article Authors and year of publication	Country	Method	Sample	Institution Size child: caregiver rates	Instruments	Measures
1. Bakermans-Kranenburg et al. (2011)		Ukraine	Case–Control	37 (18 Institution/19 family) 3–6 years old 50 % male 50 % female	Up to 200 children “High” child to caregiver ratio	1. SSP (Ainsworth et al. 1978; Cassidy and Marvin 1992) Mac Arthur coding system 2. Indiscriminate Friendliness Interview (Chisholm 1998) 3. SON-R for cognitive development 4. DNA samples for genotyping	Attachment Styles Indiscriminate Friendliness behaviour Interaction with genotype and type of care
2. a. BEIP (Bucharest Early Intervention Project)	a) Zeanah et al. (2005)	Romania	Case–Control^a^ (For report in this article)	145 (95 institution/50 community) 12–31 months 77 male 68 female	12:1 Child to caregiver ratio	1. SSP (Ainsworth et al. 1978) 2. Attachment formation rating (Carlson 2002) 3. DAI (Smyke and Zeanah 1999) 4. ORCE (NICHD 2005) adapted. To assess caregiving environment 5. Bayley Scales and ITSEA for cognitive abilities and behaviour problems	Attachment Styles Attachment formation Attachment Disorders Correlations between attachment and quality of caregiving
b. BEIP	b) Smyke et al. (2010)	Romania	Randomised Control Trial	187 (68 institution/68 Foster Care/51 community) After drop off and exclusions total number 148. 6 to 31 months at recruitment 42 months at assessment	12:1 Child to caregiver ratio	1. SSP (Ainsworth et al. 1978; Cassidy and Marvin 1992) Mac Arthur coding system 2. Bayley Scales BSID-II (1993) 3. ORCE (NICHD 2006) adapted. To assess caregiving environment	Attachment Styles Organization and security of attachment Correlations between attachment and quality of caregiving Effects of age at placement and type of it
c. BEIP	c) McLaughlin et al. (2012)	Romania	Randomised Control Trial	136 (121 after drop off) 6–30 months (assessments at entry, 42 and 54 months) 68 each gender	No information in this paper (but refers to BEIP)	1. SSP (Ainsworth et al. 1978) at baseline and with Mac Arthur (1992) coding system for pre-schoolers 2. PAPA (Egger et al. 1999) for psychiatric disorders	Attachment Styles Presence of Psychiatric disorders Gender differences
d. BEIP	d) Bos et al. (2011)	Romania	Randomised Control Trial	136 children (half of them remained in IC and other half to FC) 6–31 months at beginning Follow up at 30, 42 and 54 months of age	Institutional Foster Care specially designed for this study	1. SSP (Ainsworth et al. 1978) at baseline and with Mac Arthur (1992) coding system for pre-schoolers 2. Disturbances of Attachment Interview	Attachment Styles Attachment Disorders Other (emotional Reactivity, Brain Development, Psychiatric Morbidity)
3. Bernier et al. (2004)		USA	Longitudinal	24 Foster Children and their carers 1.5–9 months at baseline. 12–22.6 months at follow up 14 male/10 female	Foster Care	1. SSP (Ainsworth et al. 1978) 2. Parent Attachment Diary (Stovall and Dozier 2000)	Attachment Styles Associations between child’s initial attachment behaviours and attachment styles at follow up Effects of age at placement
4. a. Cole, S.	(a) 2005 (Feb.)	USA	Cross sectional	46 children and their carers 10–15 months	Foster Care	1. SSP (Ainsworth et al. 1978) 2. Caregiver Interview Form CIF (Wells and Guo 1999), including: Infant Toddler Symptom Checklist, Minnesota Infant Development Questionnaire, Support Function Scale, Parenting Stress Index and HOME Inventory for quality of care. All imbedded 3. Childhood Trauma Questionnaire	Attachment Styles Caregiver’s Factors affecting attachment Quality of care factors affecting attachment
b. Cole, S.	(b) 2005 (Dec.)	USA	Cross Sectional	46 Foster children and their caregivers. 10 to 16 months 21 male/25 female	Foster Care	1. SSP (Ainsworth et al. 1978) 2. Motivations for foster Parenting Inventory (Yates et al. 1997)	Attachment Styles Motivations for Foster parenting Associations between both variables
c. Cole, S.	(c) 2006	USA	Cross Sectional Case Control?	46 infants with kin (12) and unrelated (34) carers. 10–15 months	Foster Care	1. SSP (Ainsworth et al. 1978) 2. Caregiver Interview Form CIF (Wells and Guo 1999), including : Infant Toddler Symptom Checklist, Minnesota Infant Development Questionnaire, Support Function Scale, Parenting Stress Index and HOME Inventory for quality of care. All imbedded 3. Childhood Trauma Questionnaire	Attachment Styles
5. Dozier et al. (2001)		USA	Cross Sectional	50 children and their carers 12–24 months 29 male/21 female	Foster Care	1. SSP (Ainsworth et al. 1978) 2. AAI (George et al. 1996)	Attachment Styles Caregiver’s state of mind Relationship between attachment in children and caregivers Effect of age at placement
6. Eulliet et al. (2008)		France (Foster) Chile (adoption)	Cross Sectional	36 Foster Children 25 Adopted Children 1.6–5.6 years old 15 male/21 female 12 male/female (adopted)	Foster Care and Adopted	1. ASCT (Bretherton et al. 1990) CCH Q-Sort (Miljkovitch et al. 2003)	Attachment Styles Narratives characteristics (Expression of emotions, symbolic distance, etc.) Effect of age at placement
7. Howes and Segal (1993)		USA	Cross Sectional	16 children 8 caregivers 16–36 months 8 male 8 female	“Small” size (no information on number) 3/4 .5 :1 Child to caregiver ratio Low staff turnover	1. AQS Attachment Q set (Waters and Deane 1985) 2. Arnett Scale (1989) for caregiver’s sensitivity	Attachment Styles Sensitivity of caregivers Time in placement as mediating factor
8. Katsurada, E.		Japan	Case–Control	32 (16 institution/16 family) 4–6 years old 12 male 20 female	No information	1. Attachment Doll Play Classification System George and Solomon (1995) of the Bretherton et al. ASCT	Attachment Styles
9. Muadi et al. (2012)		R.D. Congo (Kinshasa)	Case–Control	84 (42Institution/42 family) 4– 7 years old	10:1	1. ASCT (Bretherton et al. 1990) CCH Q-Sort (Miljkovitch et al. 2003) 2. Thematic Analysis	Attachment Styles Other factors mediating attachment
10. Moore and Palacio-Quintin (2001)		Canada	Cross Sectional	26 children 14–18 years old 16 male/10female	Foster Care	1. IAPA (Armsden and Greenberg 1987) 2. A-COPE (McCubbin and Pallerson 1983)	Attachment Security/Insecurity with Foster parents and Biological parents. Comparisons Relation to coping capacities
11. Ponciano Leslie (2010)		USA	Cross sectional	76 child-foster carer dyads 9–39 months	Foster Care only	1. AQS Attachment Q-Sort (Waters and Deane 1985) 2. Maternal Behaviour Q-Sort (Pederson et al. 1990) 3. Carer Interview	Attachment Styles Maternal Sensitivity Adoption Status and Foster mother experience
12. Shechory and Sommerfeld (2007)		Israel	Cross Sectional	68 6–14 years old 47 male 21 female	No information	1. Attachment Style Classification Questionnaire (Hazan and Shaver 1987) adapted 2. CBCL for behavioural assessment	Attachment Styles Aggressive behaviour Effect of Home leaving age
13. Vorria et al. (2003)		Greece	Case-Control	128 children (86 institution/42 family) 65 caregivers 11–17 months 63 male 64 female	100 children 4/6:1 Child to caregiver ratio	1. SSP (Ainsworth et al. 1978) 2. CCTI (Plomin and DeFries 1985) for temperament 3. Bayley Scales BSID-II (1993) for cognitive development 4. PCIS (Farran et al. 1986) for maternal sensitivity 5. McCartney (1996) observational coding system. For social behaviour 6. ECERS (Harms and Clifford 1980) for quality of care	Attachment Styles Sensitivity of caregivers Quality of care Cognitive Development Temperament Socio emotional behaviour Relationship between variables

^a^Although BEIP study had a RCT design, this article reports measures for institutionalised and community children at baseline. Thus it is classified as case–control

More than half of the studies (n = 7) had a cross sectional **design**, four were case–control comparing institutionalised with family raised children, only one used a randomised control trial design (BEIP) and only one had a longitudinal design (Bernier et al. 2004).

Children’s **ages** varied widely across the studies (6 months–18 years old) making the results difficult to compare. More than half had samples with children younger than 36 months (n = 8), yet no study had exactly the same age range as another. Four other studies had samples of 3–7 year olds with little variation between them, and two considered older children (one 6–14 years; one adolescent sample).

The **measures** of attachment also varied widely, as expected given the variation in ages. Half of the studies used the Strange Situation Procedure (SSP, Ainsworth et al. 1978), but with different coding systems according to the age of the sample. Three studies used the Attachment Story Completion Task (ASCT; Bretherton et al. 1990), but one of the three considered only three of the stories (George and Solomon 1995). A further two studies used the AQS (Waters and Deane 1985) and the remaining two studies used different measures (Table 1).

All the studies reported results in terms of rates, percentage or number of children classified in the different Attachment Styles (as this was considered an inclusion criteria). However, studies varied in the number of categories considered, with some of them reporting only secure/insecure rates, while others considered the distribution across the four main categories ABCD (Avoidant, Secure, Anxious-ambivalent and Disorganised). Most of the studies describe some *factors affecting attachment,* such as age at placement, type of placement, characteristics of the caregivers (motivation, sensitivity, state of mind, childhood trauma), genetic mediators, and quality of caregiving. Some studies include measures in other areas (i.e., cognitive development, psychiatric morbidity).

### Overview of Findings

For a summary of main findings in each study plus reports on the limitations and Quality Scores (QA), see Table 2, with specific rates of attachment styles listed in Table 3 (institutional care) and Table 4 (foster care).

**Table 2:** Main findings regarding attachment, limitations and QA

Study	Main results										Limitations/possible bias	QA (%)
1. Bakermans-Kranenburg et al. (2011)	Institutional sample: 10 (55.5 %) Avoidant 5 (27.7 %) Secure 0 (0 %) Resistant 3 (16.6 %) Insecure other No significant main effect of type of care or genotype in continuous attachment disorganisation Interaction between 5HTTLPR and type of care significantly predicted attachment disorganisation (SS or SL genotype in Institutionalised children										Small sample size/sub groups Quasi-experimental design Other confounds (conditions previous to institutional care, mothers were substance users)	62.5
2. a. BEIP (Bucharest Early Intervention Project) Zeanah et al. (2005)	Institutional sample 18.9 % secure (74 % control), 3.2 % avoidant (4.0 % c), 0 % resistant (0 %), 65.3 % disorganized (22 %) 12.6 % unclassifiable 22 % of children in institutions had organized attachments strategies with their favourite caregiver (78 % of community children had) 12.6 % of institutionalized children showed so little attachment behaviour that were deemed “unclassifiable” No relation between length of institutionalization and signs of RAD No differences between the organized and disorganized children in relation to the quality of Caregiving but significant differences with the “unclassified” group who received poorer quality of care The only measure that significantly predicted attachment rating (0–5) in institutionalized sample was quality of Caregiving. Also associated with the organization of attachment In the institutionalized group only, quality of Caregiving was associated to RAD inhibited scores but unrelated to RAD disinhibited scores										In Scale for attachment formation, they propose a “tentative” cut off point Institutions with poor child caregiver ratios may be not representative of institutions in another countries Cross sectional design Coders not completely blind	70
b. BEIP Smyke et al. (2010)		CAU (I)				FC				Community	Foster Care program especially designed. May be not representative of other foster care Institutional characteristics (same as a) Assessment at 42 used a different coding system than at baseline (and variations were seen in all groups not only in FC)	75
	Secure	17.5				49.2				64.7		
	Avoidant	24.6				19.7				11.8		
	Ambivalent	12.3				8.2				13.7		
	Disorg.	5.3				13.1				9.8		
	Insec. other	40.4				9.8				0		
	No gender differences in classification but in FC sample more girls were organised at 42 months Main effect of group for security ratting (first community, then Foster Care and finally CAU/Institutional sample) No associations to Quality of Caregiving Foster Family placement causally related to improvement in children’s attachment status											
c. BEIP McLaughlin et al. (2012)	Same as BEIP b. but presents gender differences at 42 months: Females FC 63.3 % and IN 12.1 % secure (*p* < .001*) Males FC 35.3 % and IN 20.7 % secure (*p* = .205) Boys and girls with secure attachment had lower levels of internalising symptoms.										Characteristic of institutions (as previous) and Foster Care program limits generalisation of results	75
d. BEIP Bos et al. (2011)	Secure attachment: 65 % Never Institutionalised, 49 % Foster Care 18 % in Care as Usual Institutional Fewer signs of inhibited RAD in FC and NI Significant differences between groups in disinhibited RAD only at 42 months Indiscriminate Behaviour more common in Institutionalised, followed by FC and lastly NI Placement in FC before 24 months increased security in attachment and the earlier children were placed, the more organised their attachment was										Characteristic of institutions (as previous) and Foster Care program limits generalisation of results	75
3. Bernier et al. (2004)	Attachment in Foster Care: 45.8 % Secure 4.2 % Avoidant 8.3 % Resistant 41.7 % Disorganised Age at placement: Less security when placed older Older children displayed less proximity and less contact maintenance Inconsistency in child’s initial attachment behaviours immediately after placement predicted the development of a disorganised attachment Secure attachment behaviours at placement positively related to proximity seeking in SSP Avoidant behaviours in first days negatively related with contact maintenance in SSP										Small sample size Mother reported child initial behaviours (not direct observation)	73
4. a. Cole, S. 2005 (Feb.)	Attachment in Foster Care: 67 % Secure 4.3 % Insecure Avoidant 0 % Ambivalent 28 % Disorganised/Disoriented/Cannot classify Caregiver’s Trauma as negative predictor for security of attachment. Learning materials as positive predictor for security of attachment Caregiver’s sensitivity as negative predictor (over-involvement)										Self-selected sample. No information about those that refused to participate (only 69 of 172 agreed, 48 completed) Relatively small sample size Caregiver’s Sensitivity was measured using a sub scale of HOME inventory and not a specific instrument	77.2
b. Cole, S. 2005 (Dec.)	Attachment in Foster Care (same as reported in previous article a), same sample). Foster Caregiver’s Motivations are related to Infant’s Attachment: Positive predictors for secure attachment were: Desire to increase family size (significant *p* = .031) and social concern for caregiver’s specific community Predictors for Insecure attachment were: spiritual expression, desire of adoption and replacement of a grown child										Self-selected sample (as previous) Retrospective design (memory about initial motivations can change)	72.7
c. Cole, S. 2006	Attachment in Foster Care (same as reported in a) but analysed differences between kin and unrelated FC:										Potential impact of uneven sample size (n = 12, n = 34) Small sub group sample sizes	70.8
				Kin (%)				Unrelated (%)				
	Secure			67				68				
	Insecure			8				3				
	Disorganised			25				28				
5. Dozier et al. (2001)	Attachment in Foster Care: 52 % Secure 6 % Avoidant 8 % Resistant 34 % Disorganised Significant association between caregiver’s state of mind and infant attachment Non autonomous and dismissing Foster Mothers tended to have children with disorganised attachment Secure/Autonomous Foster Mothers tended to have secure children										Older children assessed with SSP (but separate analysis were conducted) Relatively small sample size	72.7
6. Eulliet et al. (2008)	Attachment in Foster Care: 69.4 % Secure 30.6 % Avoidant 0 % Hyper activated 0 % Disorganised No main effect of age at placement										Small sample size No information about sample method No information about double coding or blindness of coders to child status	62.5 %
7. Howes and Segal (1993)	Attachment in Institutional Care: 47 % Secure 44 % Avoidant 9 % Ambivalent (No measure of disorganised) Security in attachment associated with sensitivity of Caregiver Length of placement positive association with security of attachment (*p* < .01) (Institution with indicators of good quality of care)										Small sample size Majority of children in sample had previous placements No double coding for children in the study	63 %
8. Katsurada, E.	Attachment in:										Small sample and sub groups Sample method not clearly stated No double coding, no IIR Information about the measure used is not clear In FR sample the high percentage of disorganised (refused to elaborate a story) could be related to confound factors in assessment	50 %
					Institutions (%)		Family reared (%)					
	Secure				0		31.3					
	Avoidant				25		12.5					
	Ambivalent				25		25.0					
	Disorganised				50		31.3					
9. Muadi et al. (2012)	Attachment in:										No detailed information about sampling method and drop out No information about institution beyond the fact that there are one of the “best reputed”	62.5
					Institution (%)		Control (%)					
	Secure				33.3		66.7					
	Insecure Avoidant				4.7		4.7					
	Insecure Ambivalent				14.3		16.6					
	Disorganised				47.6		11.9					
	A factor of Resilience that can promote secure attachment is the establishment of a significant relationship											
10. Moore and Palacio-Quintin (2001)	Attachment in Foster Care to multiple figures 55.5 % Secure with Foster Mother (n = 10 out of 18) 45.5 % Insecure with Foster Mother (n = 8 of 18) 63.1 % Secure with Biological Mother (n = 12 of 19) 36.8 % Insecure with Biological Mother (n = 7 of 19) Attachment to fathers was less secure than attachment to mothers with both biological and foster figures Attachment with mothers was more secure with the biological mother and attachment with father was more secure with the foster figure. However other data presents more positive representations of Foster mothers in comparison to biological parents 6 Adolescents had the same patterns with biological and foster figures and 8 changed their patterns (2 of them building more secure ones with Foster Care and 4 of them more insecure ones) Security in attachment correlates with coping capacity										Small sample size Sample characterised by having regular contact with biological parents, this limits generalisation Evaluation of attachment representations only based in the Adolescent’s report in a Likert scale All information processed by researcher no inter reliability Rates of attachment not clearly presented and contradictory information	50 %
11. Ponciano Leslie (2010)	Attachment in Foster Care: 58 % Secure 11 % Avoidant 9 % Ambivalent/Resistant 22 % Unclassifiable Maternal Sensitivity: More sensitive FC had more securely attached children Less experienced Foster Mothers tended to have more securely attached children Security in attachment was higher in those children whose FC had decided to adopt them Number of children in Care in same house negatively related to attachment security Age was inversely correlated with attachment security Visit from the biological parents were inversely correlated with attachment security										No information about parents that declined participation (self-selection) All measures coded by researcher Most measures based in Foster carer’s perceptions	86
12. Shechory and Sommerfeld (2007)	Attachment in Institutional Care: 39.7 % Secure 25.0 % Avoidant 26.5 % Anxious/Ambivalent 9 % Unclassified Main effect of attachment style in Anxiety/Depression scale The aggression levels were higher for children removed before 7 years old with an insecure attachment but lower for children removed at same age but with secure attachment										Only one institution No information about quality of care provided or characteristics of the institution Sample with majority of children with Attention deficit disorder or learning disabilities	59 %
13. Vorria et al. (2003)	Attachment in:										Potential impact of uneven sample size (N = 86, N = 42) Sample method not clearly stated Control sample not representative of general population. And had low quality day care Moderate inter-ratter reliability for SSP Institution with indicators of low quality of care can affect generalisation of results	70.8
			Institution (%)						Community (%)			
	Secure		24.1						40.6			
	Avoidant		2.5						9.4			
	Ambivalent		7.6						25.0			
	Disorganised		65.8						25.0			
	Sensitivity in Caregiver’s was significantly different between groups in appropriateness and quality No correlation between attachment quality and Caregiver’s sensitivity or length of relationship

**Table 3:** Distribution of attachment styles in children living in institutions

Country/Age	Attachment style					Instrument	QA (%)
	Secure	Avoidant	Ambival	Disorg	Other		
Greece 11–17 m	**24.1**	**2.5**	**7.6**	**65.8**	–	SSP	70
Romania							
(a) 12–31 m	**18.9**	**3.2**	**0**	**65.3**	**12.6**	Strange Situation Procedure (SSP)	70
(b) 42 m (follow up)	**17.5**	**24.6**	**12.3**	**5.3**	**440.4**	SSP (Mac Arthur)	775
USA 16–36 m	47	44	9	–	–	Attachment Q-Set (Waters and Deane)	63
Ukraine 3–6 years	27.7	55.5	0	27.7	16.6	SSP (Cassidy-Marvin/Mac Arthur) and Scale for disorganised behaviour	62.5
R.D. Congo 4–7 years	33.3	4.7	14.3	47.6		Attachment Story Completion Task ASCT (CCH)	62.5
Israel 6–14 years	39.7	25	26.5	–	9.0	Attachment Style Classification Questionnaire (Hazan Shavers)	59
Japan 4–6 years	0	25	25	50	–	Attachment Doll Play-ASCT (George and Solomon 1995)	50

**Table 4:** Distribution of attachment styles in children living in foster care

Country/Age	Attachment style					Instrument	QA (%)
	Secure	Avoidant	Ambivalent	Disorganiz.	Other		
USA 9–39 m	**58**	**11**	**9**	**–**	**22**	Attachment Q-Sort (Waters and Deane)	86
Romania 42 m	**49.2**	**19.7**	**8.2**	**13.1**	**9.8**	SSP (Mac Arthur)	75
USA 10–15 m	**67**	**4.3**	**0**	**28**	–	SSP	75
USA 6–22 m	**45.8**	**4.2**	**8.3**	**41.7**	–	Parent Attachment Diary/SSP	73
USA 12–24 m	**52**	**6**	**8**	**34**	**–**	SSP/AAI	72.7
France 3–5 years	69.4	30.6	0	0	–	ASCT (CCH)	62.5
Canada 14–18 years	55.5	–	–	–	45.5 insecure	Inventaire d’Attachement Parent-Adolescent	50

#### Attachment Styles in Institutional Care

Overall, the distributions of the different attachment styles in children living in institutions have been shown to have lower rates of secure and higher rates of disorganised attachment than those observed in children living with their biological parents in the general population (Bakermans-Kranenburg et al. 2011; Katsurada 2007; Muadi et al. 2012; Zeanah et al. 2005). Table 3 summarises the distribution of attachment styles in the eight papers reporting seven studies of children living in institutions. Results show wide differences between studies, the mean rate of secure attachment was 26 % (median = 25.9, range 0–47 %), avoidant 23 % (median = 24.8, range 2.5–55.5 %), ambivalent 11.8 % (median = 10.6, range 0–26 %) and disorganised 43.6 % (median = 48.6, range 5.3–65.8 %). The high rates of disorganised attachment in children living in institutions may be a response to conditions that hinder the construction of an organised attachment. As suggested by some authors, the disorganisation in attachment patterns in these settings may not reflect the same processes as in family settings (where parental abuse or a carer’s unresolved status due to loss or trauma may be the key). In institutions, disorganised attachment may just reflect the lack of opportunity for the formation of an organised attachment due to the limited resources, such as single caregiver for many children, the shift system and staff changes (Bakermans-Kranenburg et al. 2011).

The Howes and Segal (1993) study found higher rates of attachment security compared to other studies. Notably, the institution in this study appeared to be of good quality and stability of caregiving (good child: caregiver ratio, low staff turn-over, small size), which may explain the higher secure attachment. This is consistent with results shown in the main intervention study, conducted by St. Petersburg-USA intervention project (2008). It also reflects the fact that institutions can vary widely in their quality of care and that these variations can have a strong impact on emotional development and attachment. Thus, not all institutions are the same and have the same outcomes.

#### Attachment Styles in Foster Care

In the case of foster care children (Table 4), regardless of quality, all papers except one (Eulliet et al. 2008) found that the distributions of attachment patterns are half way between institutionalised and community children when compared to control samples or general rates of attachment. The mean rate of secure attachment was 56.7 % (median = 55.5, range 45.8–69.4 %), avoidant 12.6 % (median = 8.5, range 4.2–30.6 %), ambivalent 5.58 % (median = 8.5, range 0–8.3 %) and disorganised 23.3 % (median = 28, range 0–41.7 %) (Bernier et al. 2004; Cole 2005a, b, 2006; Dozier et al. 2001; Moore and Palacio-Quintin 2001; Ponciano 2010; Smyke et al. 2010).

Three studies appear to be particularly well suited for comparison, as they have samples of similar age and country, and used the same instrument and coding system, i.e., the SSP (Bernier et al. 2004; Cole 2005a, b, 2006; Dozier et al. 2001). Within these three studies, rates of attachment also varied (i.e., disorganised attachment ranged from 28 to 41.7 %). However, communication with an author revealed that two of the studies shared some of the same sample (Bernier et al. 2004; Dozier et al. 2001); notably, these two had a smaller variation, whilst the third study (Cole 2005a, b, 2006) was quite different. Therefore, the differences may well be methodological.

In summary, the studies in both institutional and foster care have been conducted with different methodologies, with large variations in age range, instruments and the categories of attachment that are included. For these reasons the results cannot always be compared. Furthermore, the levels of deprivation in different institutions and countries can also vary considerably as can the quality of foster care programs making generalisations of conclusions very difficult. Despite this, it is notable that the studies seemed to show a pattern between institutionalised (low rate of secure attachments), foster care (mid-range) and children at home (highest rate of secure attachments).

As a whole, these findings support hypotheses 1 and 2 regarding differences in attachment styles between children raised in biological families, institutions and foster care. As expected, children in institutions develop less secure and more disorganised attachments than those raised in biological families and children living with foster families show levels of security and disorganisation in between the other two groups. However, very few studies consider samples of all these three groups—so comparisons are made with children from different countries and, thus, are limited.

#### Factors Affecting the Quality of Attachment

Supporting hypothesis 3, some studies have shown important factors mediating the quality of attachment in institutionalised and foster care (Table 5), these include:

**Table 5:** Factors affecting the quality of Attachment

Factor	Studies describing that factor is related to attachment security	Studies describing No. relation to attachment security
1. Age at placement	**2** (−), **11** (−)	3*,5*, 6,7*, 13* *studies with all children placed before 24 months
2. Number of previous placements		7
3. Length of time in placement	7(+)* *Indicators of good quality of care	13* *Indicators of low quality of care
4. Gender	**2b** **−** c* (+) *Girls in response to change from institutional to Foster care	**13**
5. Genetic Factors	1* *In interaction with type of care	
6. Adoption Status	**11** (+)	
7. Contact with Biological Parents	**11** (−)	
8. Organisation of Foster Home and Learning Materials	**4a** (+)	
9. Quality of Caregiving	**2a** (+)* *At baseline	2b* *At follow up, had changes in caregiver
10. Number of Children in Foster Care Home	**11** (−)	
11. Caregiver’s characteristics		
a. Sensitivity	**11** (+), 7 (+), **4a** (−)* *sample of children with medical fragility	13* *Caregivers with low sensitivity scores
b. Childhood trauma	**4a** (−)	
c. State of Mind	**5** (+)	
d. Motivation	**4b**	
e. Experience	**11** (−)	

Numbers in bold are studies with QA 70 % or more

Signs in brackets describe if the relationship between factor and attachment style is positive (+) or negative (−)

ID number of studies according to number used in Tables 1 and 2 for each study

##### Age at Placement

Ponciano (2013; highest quality score 86 %), found a significant correlation between age and security of attachment in a sample of Foster Care children aged 9–39 months, with younger children having higher security scores (Ponciano 2010). Similar findings were reported in BEIP: *age at placement* was a factor that mediated the quality of attachment, with more children placed in foster care before 24 months having secure attachments that those placed after that age. Also, the younger the children were when placed in foster care the higher the possibility of them developing an organised attachment (secure or insecure) at 42 months (Bos et al. 2011). These findings support the idea of flexibility and change in attachment at least during the first years of life.

Notably, most of the studies that reported no differences in attachment according to age at placement had samples with an age range of less than 24 months. For example, in the study conducted by Bernier et al. (2004; QA 73 %), attachment classifications of fostered children did not vary with age at placement. However, all participants in this study were infants placed with their caregivers between 6.5 and 19 months of age. Interestingly, children that were older at placement showed less proximity seeking and less contact maintenance in the Strange Situation Procedure than children placed earlier (Bernier et al. 2004). Similar findings were reported by Dozier et al. (2001) in the USA (age at placement: birth to 20 months); by Vorria et al. (2003) in a Greek study (age at placement 11–17 months); and in the Howes and Segal study conducted with 16 children aged 16–36 months old but where most were placed under 24 months old (M = 18.1, median = 16.5). Therefore, there appears to be a sensitive period of the first 24 months, but with later placements potentially having a negative impact on security of attachment.

The exception is the Eulliet et al. (2008) study, which did not find any significant differences in attachment security according to age of placement. In this study of 36 foster children aged 3.6 to 5.6 years old (mean age at placement = 22.2 months, SD = 15.06), 88 % of children placed in foster care between 13 and 24 months old had secure attachments to 64 % of children placed after 25 months. However, this difference did not reach statistical significance. Notably, in this study, the sample age was older and they had lived with their foster families for a longer period so other confound factors (e.g., quality of care or characteristics of caregiver) rather than age at placement, could be present and have a stronger impact on attachment security.

##### Number of Previous Placements

Only Howes and Segal (1993) reported on the effect of *number* of previous placements on quality of attachment, finding no significant effect. However, all children in this sample had at least one previous placement so no comparison could be made with children having single placements.

##### Length of Time in Placement

Time did have a significant positive relationship with security of attachment in the Howes and Segal (1993) study so the longer children were there the more likely they were to have a secure attachment. Importantly, though, in this case the children’s home was small, had very low staff turn-over and the child caregiver ratio was 3:1, all of which can be described as indicators of good quality of care. In another study, no significant differences were found regarding length of placement and attachment security; this study was conducted in a large institution described as having low quality of care (Vorria et al. 2003). Therefore, it could be hypothesized that length of placement can have a positive relationship with security on attachment in institutions that provide stability and high quality of care that may favour the formation of a secure attachment but that this does not occur in larger and more deprived institutions.

##### Gender

No significant differences were found between gender and attachment style (secure/disorganized) by Vorria et al. (2003). However, the BEIP project in Romania found that gender could be a moderating factor to the effects of placement in foster care after institutionalisation, with girls responding in a more positive way to the change in type of placement than boys (McLaughlin et al. 2012). Specifically, boys with secure attachment did not differ at 42 months between Foster Care and Care as Usual (institutional) groups, so their attachment styles tended to be more rigid.

##### Genetic Moderating Factors

In the one study to consider this, no significant main effect was found (Bakermans-Kranenburg et al. 2011). Although an interaction was established between the type of care (institutional vs. family) and genetic moderation factors, with a protective factor of the 5HTT*/*allele genotype for high scores on attachment disorganisation in institutionalized children, the authors noted that it is not clear if genetic factors can protect some children in adverse environments or if the experience of being raised in these environments can alter the expression of the gene.

##### Adoption Status

In a study with a high quality score (86 %) conducted with a sample of foster children (Ponciano 2010), significant differences in attachment security were described between children whose foster mothers had made the decision to formally adopt them and those who did not. The children with adoption status showed higher levels of security in attachment. However, the explanation for this difference can vary widely as potentially a better relationship could have motivated the desire of adoption. No information was given about the timing and reasons for the decision to adopt the foster child (Ponciano 2010). This factor needs to be studied further as in another study the motivation for adoption was found to be negatively related to security in attachment (Cole 2005b). Furthermore, motivation for adoption and adoption status (as a decision informed to the court) are possibly different constructs that are related to attachment security in different ways.

##### Contact with Biological Parents

In the same study by Ponciano (2010), a significant negative correlation between visits from biological parents and security of attachment was found, with children with fewer visits from their biological parents more likely to have a secure attachment (Ponciano 2010). We can hypothesise that, in cases of severe difficulties or maltreatment, not having contact with biological parents might facilitate the establishment of a relationship with the new carers in long-term placements. From a different perspective, another reason that may be linked with this outcomes is that contact with biological parents may discourage both the child and the foster parent to get more emotionally involved as it can place biological parent in ‘first place’ differing on them the main emotional link. The continuous presence of biological parents can be a remainder that AC is a temporary situation and thus, discourage emotional involvement. However, this factor needs to be studied further: in many countries Foster Care is seen as a temporal measure and contact with the biological family is encouraged as part of the Child’s Rights.

##### Organisation of Foster Home Environment and Appropriate Learning Materials

In another study with a sample of children in foster care, the *organisation of foster home environment and appropriate learning materials* were associated with more secure attachments (Cole 2005a, b, 2006). This can possibly be related to the capacity of the caregiver to organise the environment and provide materials according to the child’s needs, also showing they are generally more responsive to children’s needs.

##### Quality of Caregiving

The BEIP study found that in institutionalised children the *quality of caregiving* significantly predicted the attachment rating and was associated with the quality of attachment. The ‘unclassified’ group (characterised by extremely low amount of attachment behaviours) had significantly lower quality of care than the other groups. However, in the 42-month follow-up, no difference in security of attachment was found in the Care as Usual group (CAUG) regarding caregiving quality (Smyke et al. 2010). This may reflect the limitation of having a single observation measure of quality of caregiving (ORCE-NICHD), particularly since some children had changes of caregiver. This is important as the ORCE-NICHD rates the observation of the child with their favourite caregiver on 5 scales (sensitivity, stimulation of development, positive regard, flat affect and detachment). Quality of Care was also assessed in the Greek study (Vorria et al. 2003). However, no associations could be made with security of attachment because all the centres (both institutions and day-care for control group) were rated as low quality. This hinders the possibility of measuring the effect of quality of care, which is a factor that has been shown to have a strong impact on attachment formation, particularly when the quality of socio-emotional interactions between Caregivers and children is considered, such as continuity, stability of caregiving and promotion of emotional involvement (St. Petersburg-USA Orphanage Team 2008).

Quality of care was also measured in the Cole study with the HOME scale (Cole 2005a, b, 2006). The relationship between attachment and total environment variable approached significance (*p* = .086) but, when analysed separately (i.e., organisation, learning materials and variety), only learning materials were significantly related to security in attachment. However, the association between attachment security and the general score provided by the HOME inventory that includes all the above variables and others related to quality of care, was not reported in the study.

##### Number of Children Living at the Foster Home

In her study with Foster Children, Ponciano (2010) found a significant correlation between the number of children living in the foster home and the security of attachment in the child, with fewer children at home facilitating the construction of secure attachments. This is concordant with the idea of the importance of an available caregiver in the formation of a secure attachment. No other study considered this variable.

##### Caregiver’s Characteristics

Several factors related to caregiver’s characteristics were studied:

##### The Caregiver’s Sensitivity

Sensitivity has been shown to be a significant factor mediating the quality of attachment both in institutionalised and foster care children. In a study carried out with 76 foster care children, foster mothers’ maternal sensitivity (measured with Maternal Behavior Q-Sort) was a direct predictor of security in attachment (Ponciano 2010). In accordance with this, in a sample of children placed in a shelter with alternative caregivers, it was observed that more children formed secure attachments with the more sensitive and less detached caregivers (measured with Arnett Scale of Teacher Sensitivity; Howes and Segal 1993). The only study that found a non-significant relationship between sensitivity of the caregiver (measured with PCIS) and attachment classification (secure vs disorganised) was characterised by a sample of institutional caregivers all of whom had low levels of sensitivity defined by quality of interactions and appropriateness (Vorria et al. 2003).

Surprisingly, one of the studies considered in this review seems to point in the opposite direction. The study conducted by Cole with a sample of infants in foster care, describes that caregiver’s sensitivity (specifically the score in the “involvement” sub scale of the HOME inventory) was a negative predictor for the security of attachment (Cole 2005a). This could be explained as a result of an excessive or anxious monitoring of the child, e.g., due to caregiver childhood trauma, medical fragility of children in the sample (all of them having medical records of prematurity or other factors) or the close monitoring by welfare systems. Alternatively, it could be a limitation of the use of a subscale of the HOME inventory as a single measure of caregiver’s sensitivity. Further studies considering sensitivity would be useful to clarify the importance of carer’s sensitivity in alternative care. All of the studies mentioned used different instruments to assess caregiver’s sensitivity, which makes results difficult to compare.

##### Caregiver’s Childhood Trauma

The presence of child abuse and neglect in the Caregiver’s childhood experience was related to a higher rate of insecure attachments in children placed in foster care, with infants 6 % less likely to develop a secure attachment if placed with a caregiver that has experienced childhood trauma (Cole 2005a). The presence of childhood trauma was higher in kinship care than in unrelated foster care. None of the studies in institutional care considered the presence of the caregiver’s childhood trauma as a variable.

##### Caregiver’s State of Mind

In a study with 50 foster mother–infant dyads, Dozier et al. (2001) found a significant association between the caregiver’s state of mind and the quality of the infant’s attachment with non-autonomous and dismissing foster mothers tending to have children with more disorganized patterns of attachment and the more secure and autonomous foster mothers having more secure children. This is coherent with the previously mentioned factor regarding the presence of childhood trauma which is related to unresolved status.

##### Foster Caregiver’s Motivation

Motivation has been shown to have an effect on the security of attachment of infants in care. Specifically, two motivations are positive predictors for secure attachment (i.e., desire to increase the family size and social concern for the caregiver’s specific community) and three other motivations are predictors of insecure attachment (i.e., spiritual expression, replacement of a grown child and desire of adoption; Cole 2005a, b, 2006). Possible explanations for this could be that in the first two cases there exists a more adult-centred relationship, based on the foster parents beliefs or needs and not on the infant’s real needs. The desire to adopt may be a negative predictor due to the desire for a stable and life-long relationship with this child but not being sure if this would be possible or if the child could be removed from their care, thereby generating anxiety and feelings of uncertainty about the future of the relationship. However, these are hypothesises and require further study.

##### Foster Mother’s Experience

The extent of fostering and its relationship with attachment was reported in a study conducted with 76 young Foster children. No significant relationship was found between foster mother certification length and security of attachment, nor was this related to number of previous foster children. However, within this sample, the majority were experienced foster Carers, with only 11 % of foster mothers having a child in care for the first time. However, when these two variables were combined in a single factor, ‘less experienced mothers’ were more likely to have children with a secure attachment. One possible explanation could be that having previous foster children can be linked to experiences of frustration and loss that can negatively interfere with the mother’s disposition in the relationship with a new child (Ponciano 2010).

It was difficult to draw conclusions about Hypothesis 4 regarding differences in attachment styles between countries and type of institutions/foster care programs. Many differences and wide variation in rates were observed in this review. However, as several factors affect quality of attachment, it can be difficult to control confounding factors. Thus, it remains unclear whether differences are due to a) the type of AC, b) cultural factors or c) quality of care regardless of the type of AC. It should be noted, however, that several intervention studies have shown Quality of Care regardless of type of AC to be relevant (Lecannelier et al. 2014; McCall et al. 2010; St. Petersburg-USA Orphanage team 2008).

There are limited studies considering samples of different types of AC in the same country. Comparisons are usually made between one type of AC sample (i.e., either Institutional or Foster Care) and the normal population, who can have different histories and characteristics. Quality of care provided is often not reported. Finally, cultural factors have not been considered in previous studies and is something that may explain some of the differences between countries, but further studies are needed in this regard.

## Discussion

### Summary of Results and Limitations

As a whole, the studies show that attachment security can be negatively affected by the experience of alternative care and that this impact is stronger for institutional settings. However, several factors mediate the impact of the experience and not all institutions or Foster Care programs have the same outcomes for children. The mediating factors are related to characteristics of the child (age, gender, genetics and age at placement), the placements (type and quality) and the Carer (sensitivity, motivations and previous experience).

There are some important limitations in the studies that have been conducted on attachment in alternative care settings. One important limitation is the presence of differences in quality of care provided (i.e., size of institution, ratios, turn-over, sensitivity of caregiver) and, as this is not always measured, could be a main confounding factor. Other important factors not always considered in the studies are age at placement and previous placements.

There are also some methodological issues regarding the design of the studies that can have an impact on the rates of attachment classification. For example, in the BEIP study conducted in Romania, only 22 % of children in the institutional care group (study A) had organised attachments at baseline. The other children were categorised using a ‘forced classification’ where a category can be assigned based on minimal displays of behaviours and even if there were no complete attachment styles. Thus, the classifications might be questioned. Notably, in the BEIP A report, at baseline not a single child in institutional care or the community sample of never-institutionalised children was classified as having a resistant style.

Another curious finding in the BEIP study (not discussed in the papers) is the dramatic reduction of disorganised attachment between baseline and 42 months in all groups (from 65.3 to 5.3–13.1 % in institutional sample groups and from 22 to 9.8 % in community sample). This huge difference could be due to the difference in the instruments used at each of the stages, as all the studies using the SSP with the original coding system in different settings report much higher rates of disorganised attachment than the pre-school Mac Arthur coding. However, if such a factor is not taken into account, this can affect the conclusions drawn about the impact of the Foster Care program in this study, which are based on the pre-post assessment measures.

More generally, another important aspect that has been discussed is the validity of the SSP in institutional settings in which children have experienced a variety of different caregivers and are used to them leaving (due to shifts) and, in many cases to different “strangers” being present at different moments (new caregivers, volunteers, etc.). Some authors have stated that a modified version of this instrument should be used in these settings, otherwise leading to confusions in the interpretation of children’s reactions (The St. Petersburg-USA Orphanage Research Team 2008). Another way of assessing this difficulty could be the consideration of the “favourite” caregiver and the use of an attachment formation rating that can provide a better idea about the meaning of the attachment classification, placing those children with low scores on attachment formation in a more “temporary” situation that could potentially be changed if they are given the opportunity to form an attachment with their Caregiver (Bakermans-Kranenburg et al. 2011; BEIP 2005).

### Implications for Research

It is important to have more longitudinal studies (although these can be difficult to conduct) and, whilst RCTs are useful, there are important ethical concerns involved. Only one study considered outcomes for Foster Care and Institutional Care together in the same country. That design should be replicated as, in some way, it controls for possible cultural factors and could make results more comparable (especially if considering a measure of quality of care). Similarly, in institutional settings, it is important to study more factors related to the Carers’ characteristics as these have been more frequently studied in foster care. Such research could provide important information for the elaboration of public policies and international recommendations.

Contact with biological parents also requires further study to better understand influence on attachment security. Many children in foster homes or institutional care (such as Children’s Homes) have regular contact with biological parents and there can be a tension between the aim of continuity in family bonds and the aim of providing good quality and stable alternative care. This factor has initially been shown to have a negative impact in attachment formation; therefore it should be further studied in order to be considered in practical recommendations.

The impact of quality of care provided in attachment security has been shown to have contradictory results, and, although it is often measured, its influence has not always been reported. Furthering understanding of the influence of QoC on attachment formation could provide important information for improvements in alternative care settings.

Finally, local research in a wider range of countries is needed. This is to consider whether there are differences in care provided by institutions and FC programs in countries other than those previously studied. The relatively small amount of research that has been conducted in less-developed countries to date (e.g., initial research in Africa) has shown cultural differences compared to Europe and the USA that are likely to be important for outcomes in children. In Latin America, no studies with a main aim of exploring attachment styles have been published, which is important to rectify. Having said that, the few studies that have indicated different characteristics of alternative care (Herreros 2009) have not necessarily been incorporated in the recent changes to public policies in that area (following the Guidelines for Alternative Care), so it is important to progress from research to policy and practice.

## Conclusions and Implications for Practice

As this review shows, several factors can mediate the quality of attachment and outcomes are not always the same. These factors should be included in programs for the development of better care both in institutions and foster care with the specific aim of facilitating the development of an attachment formation (as secure as possible) between the children and their caregivers. In particular, age at placement has been shown to have a significant relation in attachment security with a cut-off point at 24 months after which attachment security decreases with age at placement. Thus, this should be considered in early intervention programs and placements decisions. Similarly, length of placement can have a positive effect if mediated by quality of care. The aim, then, should be to provide stability in high quality placements, rather than using a series of short placements with multiple changes and the inherent negative impact on attachment formation (Garcia Quiroga and Hamilton-Giachritsis 2014). Some characteristics of caregivers that go beyond the usual assessments have been shown to impact on attachment security. Thus, these factors need to be considered in the evaluation of potential foster or institutional carers, including assessments of motivations, state of mind, sensitivity, etc. Similarly, consideration of those features in a program of continuous support for carers (e.g., with opportunities to elaborate their own childhood traumas, improve their state of mind and increase their sensitivity) may improve the likelihood of a more positive, secure child–caregiver relationship.

In conclusion, placement in alternative care is not the final stage but more the beginning of a process for children. Whilst we continue to work towards having all children living in a family home, it is important to identify ways to improve outcome for those children remaining in alternative care. Alternative carers, whether in institutional settings or foster care, need support and guidance in the process of taking care of these especially vulnerable children. Research must take a world-wide perspective of alternative care and those working to develop policies and procedures must ensure that they take account of local cultural variations.

## Footnotes

1 This study considered a comparison sample of adopted children in Chile but all of the children in the alternative group lived in France Eulliet et al. (2008).
